# Editorial: Translocation and delivery of macromolecules across the plant plasma membrane

**DOI:** 10.3389/fpls.2023.1282105

**Published:** 2023-11-03

**Authors:** Vladimir Orbović, Keiji Numata

**Affiliations:** ^1^ Citrus Research and Education Center, University of Florida/IFAS, Lake Alfred, FL, United States; ^2^ Biomacromolecules Research Team, RIKEN Center for Sustainable Resource Science, Saitama, Japan

**Keywords:** plants, plasma membrane, organelle membranes, translocation, delivery

The plasma membrane regulates entry of atoms and molecules into plant cells. The specific amphipathic nature of the plasma membrane with a thick hydrophobic layer in the middle and thin outer hydrophilic layers makes this envelope difficult to traverse. Introduction of nucleic acids, small proteins, or complexes of the two, into the plant cells across the cell plasma membrane can have multiple outcomes. This type of transient modification of plant cell’s phenotypic features turns them into biosensors of changes in the environment or resistance to biotic and abiotic stress. Transport of different types of molecules across the organellar membranes can also lead to important changes in functioning of organelles. All these modifications have the potential of improving agricultural production in the conditions of changing climate conditions.

The plasma membrane transporters mediate uptake and translocation of amino acids into the phloem (Fischer et al., 1998). Knowledge about these transporters is important because they can serve as carriers of some agrochemicals conjugated to amino acids. This will lead to improved phloem mobility, reduced usage of agrochemicals, and reduction in environmental pollution. Xiao et al. have shown that amino acid permease RcAAP1 from Ricinus can specifically transfer L-valine-phenazine-1-carboxylic acid (L-Val-PCA) conjugate across the plasma membrane. The localization of this protein in the plasma membrane of both mesophyll cells and phloem cells supports claims for its function in uptake and phloem translocation. When expressed in yeast cells, RcAAP1 increased uptake of L-Val-PCA 2.1-fold in comparison to non-transformed yeast cells. Overexpression of RcAAP1 in Ricinus seedlings led to 1.8-fold higher concentration of L-Val-PCA in phloem sap.

Inside of the plant cell is a dynamic environment where organelles move and interact with each other due to the process of cytoplasmic streaming. These movements and interactions are associated with the state of cell’s metabolism and environmental conditions. Peroxisomes and chloroplasts share several metabolic pathways such as photorespiration and are known to interact with each other. Oikawa et al have developed a semi-automatic high-throughput method to quantitatively evaluate the interactions between peroxisomes and chloroplasts using a distance transformation algorithm and high-resolution 3D fluorescent images taken by confocal laser scanning microscopy. By using this method, they measured the 3D distance between the center of peroxisome and chloroplast surface in leaf mesophyll cells of *Arabidopsis thaliana* under different light conditions. These measurements were also done in different types of cells including root cells containing plastids not performing photosynthesis and confirmed that these organelles are closer to each other in light than in dark. These results suggest that chloroplasts play a role in this interaction.

Transformation efficiency of organellar genomes in plants depends on the number of DNA carriers, the amount of DNA released, and the recombination efficiency between the organellar genome and the released DNA. The cell-penetrating peptides are not the most efficient for chloroplast genome transformation, making the recombination efficiency between the chloroplast genome and the released DNA even more important. The group lead by Oikawa et al reported on the entry of a cell-penetrating peptide-DNA complex into chloroplasts of Arabidopsis leaves. By using confocal microscopy, they observed movement of the chloroplast transit peptide-DNA (cpPD-DNA) complex across the chloroplast membranes. The cpPD-DNA complex was labeled with cyanine fluorescent dye and chloroplast membranes were stained with the FM4-64 dye. Time lapse photography allowed for establishment of the time course for the cpPD-DNA complex entry. Furthermore, the study has shown that cpPD-DNA complexes became enveloped by the chloroplast outer membrane after they reached the chloroplast surface and were gradually pulled into the chloroplast. Finally, the DNA was released from the cpPD-DNA complex.

Successful attack of plants by pathogens depends, among other things, on the ability of the pathogen to introduce effector molecules into the host cells. One of the complexes that allow such transfer is the Transport III secretion system, T3SS, that can deliver effector molecules directly into the cytoplasm of the plant host’s cells. There are multiple elements that contribute to proper assembly and functioning of T3SS of *Xanthomonas euvesicatoria* which is the causal agent of a bacterial spot disease in pepper and tomato plants. These elements include the HpaA, HpaB, and TrM proteins. In the study by Drehkopf et al, that unraveled complex interactions between these proteins, it was shown that HpaB generates a recognition site for the TrM at the T3SS system and thus restricts the access to the secretion channel to effector proteins. The TrM also interfered with the efficient interaction of HpaA with several T3S system components, suggesting that it prevents premature binding and translocation of HpaA. These results shed new light on contribution of TrM and HpaB to substrate recognition and suggest that the TrM increases the binding specificity between HpaA and T3S system components.

The research presented in this Research Topic shows complexity of molecular interactions mediating translocation of materials across membranes in plant cells. It also points to the plethora of opportunities for future research considering the knowledge that exists for animal cells but is barely present for plant cells.

## Author contributions

VO: Writing – original draft. KN: Writing – review & editing.

